# Improving HPV vaccine knowledge and acceptance through educational intervention: a quasi-experimental study of healthcare students in Saudi Arabia

**DOI:** 10.3389/fpubh.2026.1940176

**Published:** 2026-10-19

**Authors:** Lama Alzamil, Layla Faqih, Sarah Alharbi, Esraa Aldawood

**Affiliations:** Department of Clinical Laboratories Sciences, The College of Applied Medical Sciences, King Saud University, Riyadh, Saudi Arabia

**Keywords:** educational intervention, healthcare students, HPV prevention, HPV vaccine, Saudi Arabia, vaccine hesitancy

## Abstract

**Introduction:**

Human papillomavirus (HPV) is a major cause of cervical, anogenital, and oropharyngeal cancers worldwide. Despite the availability of a highly effective vaccine, HPV vaccine uptake remains notably low in Saudi Arabia. This study aimed to assess the effectiveness of an educational intervention in improving HPV vaccine knowledge and reducing hesitancy among healthcare students in Saudi Arabia.

**Methods:**

A quasi-experimental one-group pre-test/post-test design was conducted at King Saud University, Riyadh, Saudi Arabia, from May 2023 to March 2024. A total of 355 healthcare students completed a validated questionnaire before a structured one-hour educational session addressing HPV infection, associated cancers, and vaccination, and 324 post-session questionnaires were returned. Data were analyzed using descriptive statistics, chi-square tests, and logistic regression.

**Results:**

Following the intervention, correct responses to knowledge items increased from 12–22% pre-intervention to 45–80% post-intervention (all *p* < 0.0001), while vaccine hesitancy declined from 63.7 to 28.7% (*p* < 0.0001). Male gender was independently associated with higher hesitancy, whereas prior awareness of the vaccine was associated with lower hesitancy. Lack of information, the leading reason for hesitancy pre-intervention, declined markedly post-intervention.

**Conclusion:**

These findings highlight the effectiveness of targeted educational programs in improving HPV vaccine awareness and acceptance among healthcare students in Saudi Arabia, supporting their integration into healthcare curricula.

## Introduction

Human papillomavirus (HPV) is the most common sexually transmitted infection worldwide (1). It is estimated that 80% of individuals will contract the infection at least once in their lifetime (2). While most HPV infections are asymptomatic, persistent infections with high-risk HPV types, such as HPV 16 and 18, can lead to various cancers in both women and men. In 2018, approximately 620,000 new cancer cases in women and 70,000 in men were attributed to HPV globally (3). Among women, cervical cancer represents 90% of all HPV-related cancers. In men, HPV is mainly associated with cancers of the oropharynx, anus, and penis.

Studies have consistently shown that HPV vaccines are highly effective at preventing HPV infections and reducing the incidence of HPV-related cancers and associated morbidity and mortality (4–6). The vaccine has been available since 2006 and was recommended to countries by the World Health Organization (WHO) in 2017 (7). The current WHO guidelines on HPV vaccination recommend primary vaccination for girls aged 9–14 years, ideally before starting sexual activity, with boys and older females as secondary targets. The WHO has set a global target to achieve 90% HPV vaccine coverage for girls by 2030 (8).

In Saudi Arabia, the burden of HPV-related diseases remains significant, with cervical cancer ranking as the 8th most common cancer among women (9). National data reported that 358 new cases of cervical cancer are diagnosed annually with 179 reported deaths (9). Although a comprehensive national guideline for cervical cancer screening has not been fully established (9–11), the HPV vaccine was officially included in the national immunization schedule in 2019. The vaccine is recommended for girls aged 9 to 14 years, with additional recommendations for females aged 15 to 26 years as part of the adult immunization schedule (12, 13).

Despite the vaccine’s availability and its proven effectiveness, global uptake of the HPV vaccine remains low (14). In Saudi Arabia, the situation is particularly concerning, with uptake remaining critically low; studies have reported rates as low as 1%. For example, a study conducted in Riyadh found that only 1% of Saudi women had received the vaccine (15). Another study reported a 4% uptake among women in the Eastern province (16). Similar findings were reported by Gari et al., with a 4% vaccination rate among Saudi women (17), and the highest reported rates not exceeding 10% (18, 19).

Vaccine hesitancy is defined as a “delay in acceptance or refusal of vaccination despite the availability of vaccination services” (20), and is widely documented across various vaccines, including those for HPV, influenza, and COVID-19 (21), complicating efforts to control vaccine-preventable infectious diseases. In 2019, the WHO identified vaccine hesitancy as one of the top 10 threats to global health (22). Several factors have contributed to HPV vaccine hesitancy, including a lack of knowledge about HPV and its vaccine (23). In the context of Saudi Arabia, additional factors such as religious and moral concerns, unmarried status (24), beliefs of not being at risk, and not having multiple sexual partners also contribute to reluctance to accept the HPV vaccine (24–26).

The association between HPV vaccine hesitancy and insufficient knowledge is well-documented among healthcare students. Farsi et al. reported that 58.4% of male medical students were unaware of the HPV vaccine (27). Additionally, Aldawood et al. identified a high hesitancy rate of 59.1% among health college students, primarily attributed to a lack of information about the vaccine (28). Similarly, AlShamlan et al. found that poor knowledge and widespread misconceptions were the leading causes of vaccine hesitancy among female healthcare professionals (24). Alanazi et al. further highlighted this issue, revealing that many female nursing students lacked knowledge about the appropriate timing and dosing schedule required for effective HPV vaccination (29). The persistence of such knowledge gaps among future healthcare providers is particularly concerning given their essential role in public health advocacy and patient education.

Despite the growing body of literature documenting knowledge deficits and vaccine hesitancy among Saudi healthcare students, few studies have prospectively evaluated the impact of a structured educational intervention on both HPV vaccine knowledge and hesitancy within this population. Existing studies have been largely cross-sectional in design, capturing the prevalence of hesitancy without addressing its modifiability through targeted education (28, 30). This gap is particularly consequential given the pivotal role healthcare students are expected to play in future vaccine advocacy and patient counseling. The present study addresses this gap by evaluating the effectiveness of a structured one-hour educational intervention in improving HPV vaccine knowledge and reducing hesitancy among healthcare students in Riyadh, Saudi Arabia. The findings aim to inform the development of scalable, curriculum-based strategies to promote HPV vaccination.

## Methods

### Study design

We conducted a quasi-experimental one-group pre-test and post-test study to assess the effectiveness of an HPV vaccine educational program among college students in Riyadh, Saudi Arabia. The study was carried out at King Saud University, Riyadh, involving students from the Colleges of Applied Medical Sciences, Nursing, Dentistry, Medicine, and Pharmacy. Participants were recruited through non-probability convenience sampling, with invitations extended during regular class sessions. Students were informed about the study objectives and invited to participate voluntarily. Those who provided informed consent completed the pre-test questionnaire before the educational intervention and the post-test questionnaire immediately after the session. A total of 355 students met the inclusion criteria, which required participants to be full-time undergraduate students willing to participate. Students in the preparatory year or internships were excluded from the study. This study is reported in accordance with the Transparent Reporting of Evaluations with Nonrandomized Designs (TREND) statement (31).

### Intervention

Prior to developing the HPV educational package, a comprehensive review of the relevant literature was undertaken. Content was additionally informed by information available on the official Saudi Ministry of Health website. The educational intervention was developed and delivered by the study investigators, who are microbiologists and faculty members at King Saud University.

The intervention consisted of a face-to-face interactive didactic lecture supported by a Microsoft PowerPoint presentation. One educational session was conducted for each participant group. The presentation covered HPV infection, modes of transmission, HPV-associated cancers, and preventive measures, including cervical cancer screening (Pap smear), HPV vaccine dosing schedules, recommended target age groups, vaccine effectiveness against HPV-related conditions, and the optimal timing for vaccination. The lecture was delivered in English, as English is the standard language used in health colleges at King Saud University.

Following the lecture, an open discussion and question-and-answer session were conducted to address participants’ questions and clarify misconceptions. The total duration of the lecture and discussion was approximately 1 h. The same standardized PowerPoint presentation and educational content were used for all groups to ensure consistency of the intervention.

### Instrument

Data were collected using a two-part self-administered questionnaire previously developed and validated among a comparable population of Saudi healthcare students by Aldawood et al. (28). The questionnaire was administered in English, consistent with the language used in health colleges at King Saud University. The first part assessed participants’ sociodemographic characteristics, including gender, age, college, university year, nationality, smoking status, and marital status. The second part comprised seven true/false knowledge items covering key aspects of HPV vaccination, including dosing schedule, target population, efficacy against cervical cancer and genital warts, and the continued need for cervical screening following vaccination; these items were originally adapted from the internationally validated HPV knowledge scale developed by Waller et al. (32). An additional item assessed prior receipt of the HPV vaccine. Vaccine hesitancy was operationalized using a single item: participants were asked “Would you be interested in getting the HPV vaccine?” and those responding “No” or “I don’t know” were classified as hesitant, consistent with the WHO definition of vaccine hesitancy as delay in acceptance or refusal of vaccination despite the availability of vaccination services (20).

### Data collection

The study targeted students enrolled in healthcare colleges at King Saud University. Sample size was estimated using the Raosoft online calculator (Raosoft, Inc., Seattle, WA, USA), based on a 5% margin of error, a 90% confidence level, and an assumed 50% response distribution, yielding a minimum sample size of 250 participants. A total of 355 students completed the pre-test assessment, and 324 post-test questionnaires were returned (91.3%). Thirty-one students who completed the pre-test did not complete the post-test and did not provide reasons for non-completion. Questionnaires were anonymous and could be linked through the last four digits of the participant’s mobile number. When this identifier was missing or incorrectly entered, linkage was attempted using an exact match of college, gender, age, university year, and GPA within the same session. Using this procedure, 314 pre-test respondents (88.5%) were successfully linked to a corresponding post-test response. The remaining 10 students had completed both questionnaires but could not be matched due to missing or inconsistent identifiers and demographic information. Baseline characteristics of participants included and not included in the paired analysis are presented in Table 1. A comparison of participants who completed both the pre-test and post-test questionnaires (*n* = 324) with those lost to follow-up after the pre-test (*n* = 31) is provided in Supplementary Table S1. Reasons for declining participation included scheduling conflicts, limited interest in the study topic, overlapping academic obligations, and personal preferences. Data collection was conducted in classrooms immediately following students’ regularly scheduled lectures. As teaching groups in the participating health colleges typically consisted of no more than 20 students, multiple data collection sessions were required across different colleges and academic years, resulting in data collection being conducted between May 2023 and March 2024. The pre-test and post-test questionnaires were administered electronically using Google Forms. Data collection was conducted immediately before and after each educational session to evaluate the immediate effect of the intervention.

**Table 1:** Baseline characteristics of all pre-test respondents (*N* = 355) and of respondents who could and could not be linked to a post-test.

Variable	Category	Total (*N* = 355)	Linked to post-test (*n* = 314)	Not included in paired analysis (*n* = 41)	*p* value†
Gender	Female	270 (76.1)	237 (75.5)	33 (80.5)	0.563
	Male	85 (23.9)	77 (24.5)	8 (19.5)	
Age (years)	Mean ± SD	20.1 ± 1.1	20.1 ± 1.1	20.0 ± 1.0	0.605
	18–19	124 (34.9)	108 (34.4)	16 (39.0)	0.448
	20–21	194 (54.6)	171 (54.5)	23 (56.1)	
	≥22	37 (10.4)	35 (11.1)	2 (4.9)	
College	Applied Medical Sciences	287 (80.8)	257 (81.8)	30 (73.2)	0.196
	Nursing	48 (13.5)	42 (13.4)	6 (14.6)	
	Pharmacy	12 (3.4)	10 (3.2)	2 (4.9)	
	Medicine	5 (1.4)	3 (1.0)	2 (4.9)	
	Dentistry	3 (0.8)	2 (0.6)	1 (2.4)	
University year	1–2	137 (38.6)	123 (39.2)	14 (34.1)	0.571
	3	131 (36.9)	114 (36.3)	17 (41.5)	
	≥4	82 (23.1)	75 (23.9)	7 (17.1)	
GPA (out of 5)	4.0–5.0	188 (53.0)	168 (53.5)	20 (48.8)	0.026
	2.9–3.99	144 (40.6)	130 (41.4)	14 (34.1)	
	<2.9	5 (1.4)	3 (1.0)	2 (4.9)	
	Not disclosed	18 (5.1)	13 (4.1)	5 (12.2)	
Nationality	Saudi	352 (99.2)	311 (99.0)	41 (100.0)	1.000
	Non-Saudi	3 (0.8)	3 (1.0)	0 (0.0)	
Marital status	Single	353 (99.4)	313 (99.7)	40 (97.6)	0.218
	Married	2 (0.6)	1 (0.3)	1 (2.4)	
Smoking	Non-smoker	340 (95.8)	300 (95.5)	40 (97.6)	1.000
	Smoker	15 (4.2)	14 (4.5)	1 (2.4)	
Heard of HPV vaccine	Yes	124 (34.9)	111 (35.4)	13 (31.7)	0.729
	No	231 (65.1)	203 (64.6)	28 (68.3)	
Baseline vaccine hesitancy	Hesitant	226 (63.7)	200 (63.7)	26 (63.4)	1.000
	Not hesitant	129 (36.3)	114 (36.3)	15 (36.6)	

† The “not included in paired analysis” group consisted of 31 participants who completed the pre-test but did not complete the post-test and 10 participants who completed both questionnaires but could not be matched due to missing or inconsistent identifiers. Values are n (%) unless stated. GPA grade point average. *Chi-square or Fisher’s exact test for categorical variables; independent-samples t-test for mean age.

### Statistical analysis

Categorical variables (gender, age group, college, university year, GPA, nationality, marital status, smoking and HPV vaccine awareness) were summarized as frequencies and percentages, and age as mean ± standard deviation. Baseline characteristics of pre-test respondents who could and could not be linked to a post-test were compared using the chi-square or Fisher’s exact test, and the independent-samples t-test for age. Pre- and post-intervention proportions of correct responses to each knowledge item, of hesitant participants and of each reason for hesitancy were compared using the chi-square test. As a sensitivity analysis, knowledge items and hesitancy were also compared within the 314 linked pre–post pairs using McNemar’s test. Multivariable logistic regression was used separately for the pre-test and post-test data to identify factors independently associated with vaccine hesitancy (hesitant vs. not hesitant), with gender, age group (18–19 years as reference), university year (years 1–2 combined as reference), GPA (4.0–5.0 vs. <4.0) and prior awareness of the HPV vaccine as covariates. Participants who did not disclose their GPA were excluded from these models, and smoking status was not included because of insufficient variability (4.2% smokers). Results are presented as adjusted odds ratios (AORs) with 95% confidence intervals (CIs). A two-sided *p*-value < 0.05 was considered statistically significant. Analyses were conducted using GraphPad Prism 10 (GraphPad Software, San Diego, CA, USA) and Python 3.12 (statsmodels 0.14).

## Results

### Socio-demographic characteristics of the respondents

Table 1 presents the socio-demographic characteristics of the 355 pre-test respondents. The majority were female (76.1%), with males representing 23.9% of the sample. Mean age was 20.1 ± 1.1 years (range 18–24); 34.9% were aged 18–19 years, 54.6% were 20–21 years and 10.4% were ≥22 years. Most participants were from the College of Applied Medical Sciences (80.8%), followed by Nursing (13.5%), Pharmacy (3.4%), Medicine (1.4%) and Dentistry (0.8%). Nearly all were Saudi (99.2%) and single (99.4%). Participants included in the paired analysis (*n* = 314) and those not included (*n* = 41) were similar in gender, age, college, university year, nationality, marital status, smoking, prior awareness of the HPV vaccine and baseline hesitancy (63.7% vs. 63.4%; all *p* > 0.19); the only difference was a higher proportion of undisclosed GPA among participants not included in the paired analysis is (12.2% vs. 4.1%; *p* = 0.026 for the overall GPA distribution). A comparison of participants who completed both the pre-test and post-test questionnaires (*n* = 324) with those lost to follow-up after the pre-test (*n* = 31) showed that baseline characteristics were comparable between the two groups (Supplementary Table S1).

### HPV vaccine knowledge and hesitancy before and after the educational intervention

Before introducing the intervention, participants were asked whether they had ever received the HPV vaccine and if they believed an awareness program would help them decide whether to take it (Figure 1). The majority (66%) had not received the HPV vaccine, and 31% were unsure if they had received it, while only 1.6% had actually received it (Figure 1a). Most participants (79.7%) felt that an HPV vaccine awareness program would be useful in helping them decide whether to take the vaccine, while only 4.8% felt it would not be helpful (Figure 1b).

**Figure 1:**
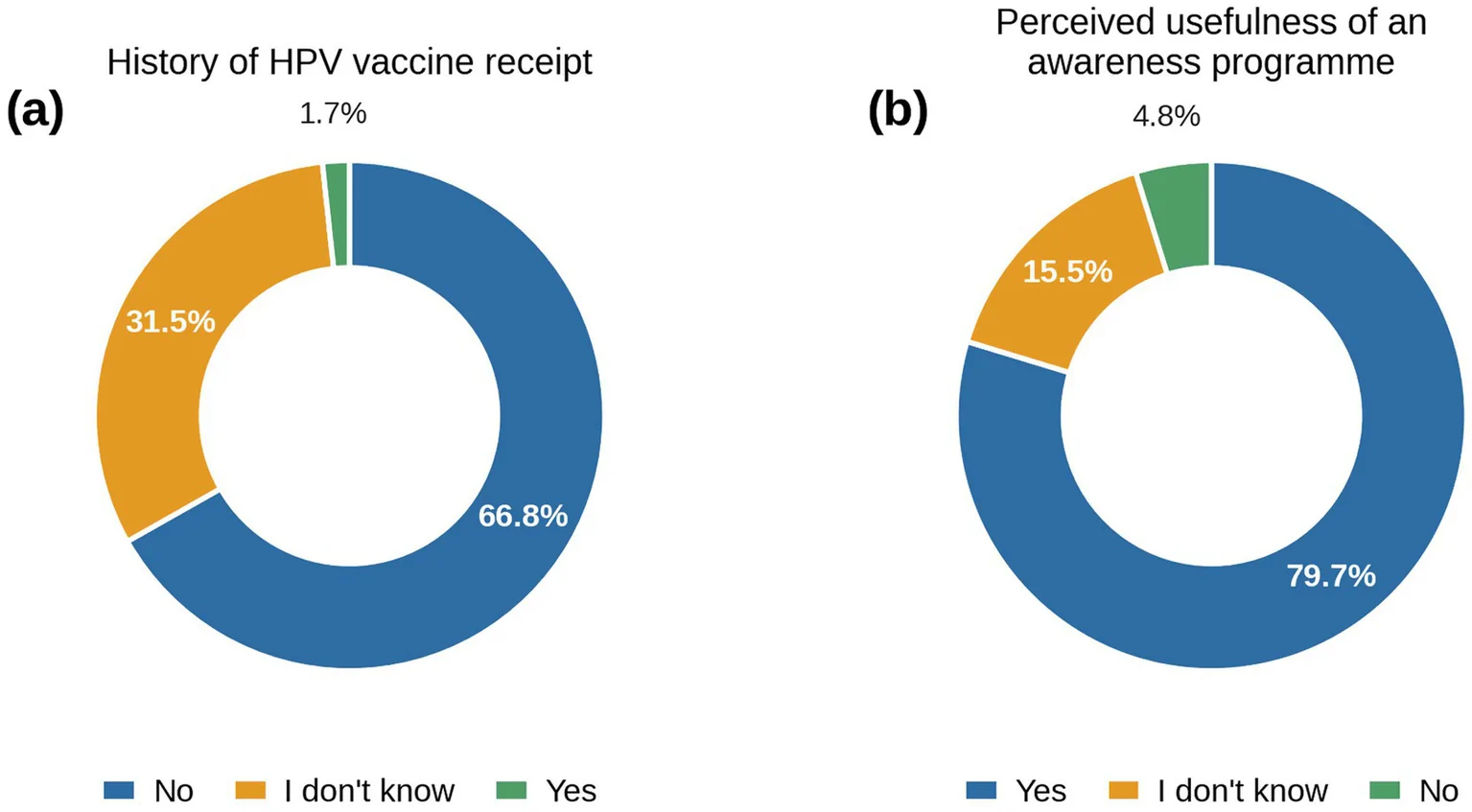
History of HPV vaccine receipt and opinions on educational program utility among participants. **(a)** History of HPV vaccine receipt among participants (*N* = 355). **(b)** Participant opinions on whether an HPV vaccine awareness program would be useful in helping them decide whether to take the vaccine.

Participants were asked seven questions to assess their knowledge about the HPV vaccine before and after the educational intervention. The results in Table 2 show that after the educational intervention, correct responses to all knowledge statements increased. Pre-intervention, correct responses ranged from 12.4 to 22.3%, while post-intervention, correct responses ranged from 45.1 to 79.6%. The highest post-intervention correct response rate was for the statement “The HPV vaccines are most effective if given to people who have never had sex” (79.6%), compared to 18.0% pre-intervention. The lowest post-intervention correct response rate was observed for “HPV vaccines offer protection against all STIs” (45.1%), compared with14.6% pre-intervention. Correct responses to the statement “HPV vaccines require three doses” increased from 12.4% pre-intervention to 76.5% post-intervention. Similarly, correct responses regarding vaccine’s protection against most cervical cancers increased from 22.3 to 79.0%.

**Table 2:** Knowledge about the HPV vaccine before (*N* = 355) and after (*N* = 324) the educational intervention.

Statement	Pre-intervention (*N* = 355)		Post-intervention (*N* = 324)		*p* value†
	Correct n (%)	Incorrect n (%)	Correct n (%)	Incorrect n (%)	
HPV vaccines require three doses	44 (12.4)	311 (87.6)	248 (76.5)	76 (23.5)	<0.0001
HPV vaccines offer protection against all STIs*	52 (14.6)	303 (85.4)	146 (45.1)	178 (54.9)	<0.0001
HPV vaccines are most effective if given to people who have never had sex	64 (18.0)	291 (82.0)	258 (79.6)	66 (20.4)	<0.0001
Someone who has had HPV vaccine cannot develop cervical cancer	57 (16.1)	298 (83.9)	152 (46.9)	172 (53.1)	<0.0001
HPV vaccines offer protection against most cervical cancers	79 (22.3)	276 (77.7)	256 (79.0)	68 (21.0)	<0.0001
One of the HPV vaccines offers protection against genital warts	65 (18.3)	290 (81.7)	249 (76.9)	75 (23.1)	<0.0001
Girls who have had HPV vaccine do not need a Pap smear test when older	64 (18.0)	291 (82.0)	243 (75.0)	81 (25.0)	<0.0001

* STIs sexually transmitted infections. Participants who reported never having heard of the HPV vaccine were routed past the knowledge items and were classified as incorrect. †Chi-square test.

Participants were then asked about their willingness to receive the HPV vaccine. Anyone who responded with “I do not know” or “No” was considered hesitant. The results depicted in Figure 2 illustrate a marked decrease in HPV vaccine hesitancy among participants following the educational intervention. Pre-intervention, 63.7% of participants were classified as hesitant about receiving the HPV vaccine, while 36.3% were not hesitant. Post-intervention, the proportion of hesitant participants dropped significantly to 28.7%, while 71.3% expressed no hesitancy toward the vaccine. This shift was statistically significant (*p* < 0.0001), underscoring the effectiveness of the intervention in reducing vaccine hesitancy. In the sensitivity analysis restricted to the 314 linked pre–post pairs, hesitancy decreased from 63.7 to 28.0% (McNemar *p* < 0.0001): 120 participants changed from hesitant to willing and 8 from willing to hesitant, and all seven knowledge items improved significantly (all *p* < 0.0001; Supplementary Table S2).

**Figure 2:**
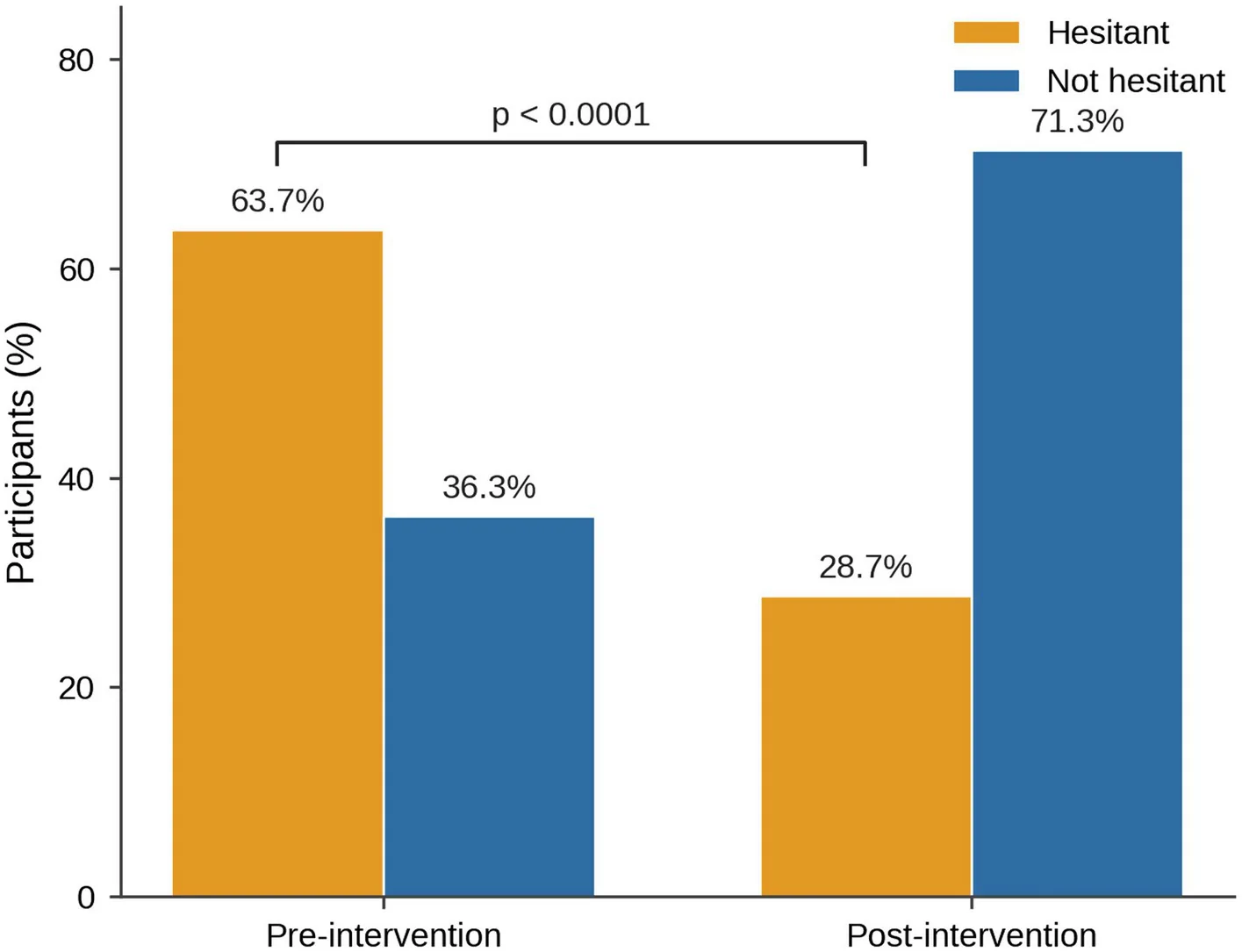
HPV vaccine hesitancy before and after the educational intervention. Participants were asked “Would you be interested in getting the HPV vaccine?” Those who responded “I don’t know” or “No” were classified as hesitant (*p* < 0.0001).

The reasons for HPV vaccine hesitancy among participants were assessed both before and after the educational intervention (Figure 3). The most common pre-intervention reason was “Not enough information,” reported by 52.4% of hesitant participants. This percentage dropped significantly to 15.1% post-intervention (*p* < 0.001), highlighting a substantial improvement in addressing the knowledge gap. Reasons such as “Worried about the safety of the vaccine” and “Doctors not recommending it” showed slight decreases after the intervention but did not reach statistical significance.

**Figure 3:**
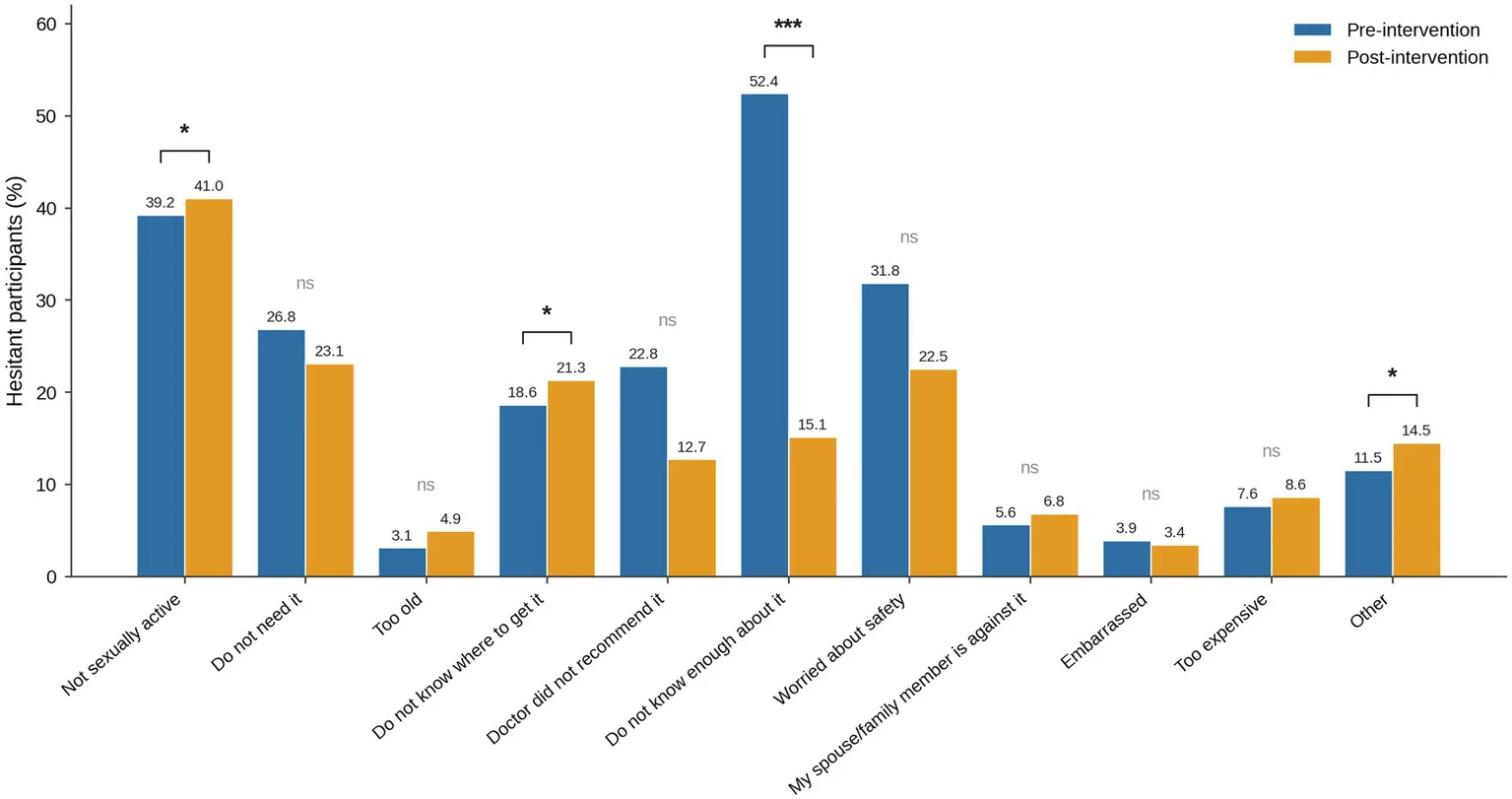
Reasons for hesitancy toward the HPV vaccine before and after the educational intervention among hesitant participants.

### Association between hesitancy and student characteristics before and after the educational intervention

In multivariable logistic regression (Table 3), male students had significantly higher odds of vaccine hesitancy than females both before (AOR 2.95, 95% CI 1.48–5.90, *p* = 0.002) and after the intervention (AOR 6.07, 95% CI 3.10–11.87, *p* < 0.0001). Prior awareness of the HPV vaccine was associated with lower odds of hesitancy both before (AOR 0.27, 95% CI 0.16–0.45, *p* < 0.0001) and after the intervention (AOR 0.40, 95% CI 0.16–0.96, *p* = 0.040). Age group, university year and GPA were not independently associated with hesitancy at either time point.

**Table 3:** Multivariable logistic regression of factors associated with HPV vaccine hesitancy before (*n* = 332) and after (*n* = 301) the educational intervention.

Characteristic	Category	Pre-intervention				Post-intervention			
		n	AOR	95% CI	*p* value	n	AOR	95% CI	*p* value
Gender	Female (ref)	251	—	—	—	218	—	—	—
	Male	81	2.95	1.48–5.90	0.0022	83	6.07	3.10–11.87	<0.0001
Age (years)	18–19 (ref)	115	—	—	—	99	—	—	—
	20–21	180	0.88	0.51–1.54	0.6647	165	0.54	0.29–1.02	0.0558
	≥22	37	0.82	0.30–2.25	0.6950	37	0.84	0.26–2.69	0.7741
University year	1–2 (ref)	129	—	—	—	117	—	—	—
	3	124	0.95	0.54–1.67	0.8523	109	0.61	0.31–1.21	0.1549
	≥4	79	0.58	0.29–1.18	0.1338	75	0.73	0.29–1.82	0.4958
GPA	4.0–5.0 (ref)	187	—	—	—	167	—	—	—
	<4.0	145	0.80	0.46–1.38	0.4140	134	1.48	0.79–2.79	0.2216
Heard of HPV vaccine	No (ref)	216	—	—	—	27	—	—	—
	Yes	116	0.27	0.16–0.45	<0.0001	274	0.40	0.16–0.96	0.0400

AOR adjusted odds ratio, CI confidence interval, GPA grade point average, ref reference category. Models include all variables shown; participants with undisclosed GPA (18 pre, 17 post) or unclassifiable university year (5 pre, 7 post) were excluded, and smoking status was omitted owing to insufficient variability. McFadden pseudo-R^2^: 0.12 (pre), 0.16 (post).

## Discussion

The results of this study underscore the significant impact of educational interventions on improving knowledge and reducing hesitancy toward the HPV vaccine among Saudi healthcare students. The findings align with previous research that identifies inadequate knowledge as a primary determinant of vaccine hesitancy, particularly in contexts where cultural, religious, and social factors influence health behaviors (20, 24). Another study found that around 40% of Saudi women refused to receive the HPV vaccine, with the most frequently reported reason being not having heard about it (17). Similarly, Alhusayn et al. reported that 89.5% of parents had neither received the HPV vaccine for themselves nor their children, primarily due to a lack of awareness about its existence (33). Moreover, vaccine hesitancy decreased substantially following the educational intervention, supporting the potential role of targeted educational initiatives in addressing this public health challenge.

The socio-demographic characteristics of the participants provide critical context for interpreting the study results. The majority of participants were female (76.06%), and most were enrolled in the College of Applied Medical Sciences (80.84%). This skew in representation reflects the composition of the student population within the participating institution and emphasizes the importance of targeting such groups in educational interventions, given their future roles in healthcare. The overwhelming majority of participants were Saudi nationals (99.15%), and nearly all were single (99.44%). These demographics suggest a relatively homogenous participant pool, which may limit the generalizability of the findings to more diverse populations.

Knowledge gaps surrounding the HPV vaccine have consistently been reported as a major barrier to vaccine uptake, not only globally (34–39), but particularly in Saudi Arabia (18, 19, 30, 40, 41). The pre-intervention findings of this study, where correct response rates to HPV knowledge questions were as low as 12.4%, mirror those of prior research (30). Additionally, previous studies in Saudi Arabia have documented a pervasive lack of awareness among both healthcare students and the general population (30, 33). The post-intervention knowledge gains, with correct responses reaching as high as 79.6%, illustrate the potential for well-designed educational programs to bridge these gaps. This improvement underscores the importance of education as a critical strategy for overcoming vaccine hesitancy, especially when cultural misconceptions and limited awareness act as compounding barriers (42).

The reduction in vaccine hesitancy observed post-intervention indicates that knowledge plays a pivotal role in shaping health behaviors. Lack of information was the most frequently reported reason for hesitancy before the intervention and showed a marked reduction afterwards, suggesting that educational interventions may help address information-related barriers. However, other reasons for hesitancy, such as concerns about vaccine safety and insufficient recommendations from healthcare providers, saw less significant reductions, suggesting the need for multi-faceted approaches that go beyond information dissemination.

The demographic analysis provided further insights. Male students exhibited significantly higher odds of vaccine hesitancy compared to females both before and after the intervention, with the AOR increasing from 2.95 pre-intervention to 6.07 post-intervention. Notably, the gender gap in hesitancy widened rather than narrowed following the intervention, a finding that merits further exploration. One plausible explanation is that the educational intervention was more effective in shifting female attitudes than male attitudes, resulting in a relatively greater reduction in hesitancy among females and thus a wider post-intervention disparity.

This differential responsiveness may reflect the gendered framing of HPV vaccination in Saudi Arabia, where the national immunization program targets females aged 9–14 and public health messaging has historically emphasized cervical cancer prevention, a disease that directly affects women (9, 12). Male students may therefore perceive the vaccine as less personally relevant, and a single educational session may be insufficient to overcome deeply held beliefs about personal risk, sexual activity, and vaccine necessity in this group (26, 27). This interpretation is consistent with prior findings from Saudi Arabia, where male healthcare students have demonstrated substantially higher hesitancy rates than their female counterparts (28), and with broader literature documenting that men are less likely to perceive themselves as susceptible to HPV-related disease (27). Furthermore, in conservative cultural contexts such as Saudi Arabia, discussions of sexually transmitted infections may carry social stigma that disproportionately inhibits male engagement with HPV-related health messaging (25). These findings suggest that generic educational interventions delivered uniformly to mixed-gender audiences may inadvertently widen existing gender disparities in vaccine acceptance, and that future programs should incorporate gender-tailored components that specifically address male-relevant risk framing, such as HPV’s association with oropharyngeal and penile cancers, and the vaccine’s role in protecting future partners.

The success of this intervention among healthcare students has broader implications for public health. By improving knowledge and reducing hesitancy among future healthcare providers, such interventions can create a ripple effect, enhancing vaccine advocacy and acceptance in the wider community, consistent with prior findings (43). Healthcare students are uniquely positioned to influence public perceptions and address vaccine misconceptions in their future roles. As future healthcare providers, they can contribute to community awareness by providing evidence-based counseling, recommending vaccination during patient encounters, addressing misconceptions regarding HPV transmission and vaccine safety, and supporting informed decision-making among patients and families. This underscores the value of integrating HPV vaccine education into healthcare curricula as a sustainable strategy for improving vaccine uptake. However, the study also highlights the limitations of education-focused interventions when addressing deep-seated cultural and systemic barriers. Concerns about vaccine safety and the lack of strong recommendations from healthcare providers remain significant issues. These findings call for a comprehensive approach that combines education with broader public health strategies, such as campaigns to normalize vaccination, stronger healthcare provider recommendations, transparent communication addressing vaccine safety concerns, community engagement, and policy measures to enhance access and convenience. The findings of this study carry meaningful implications for public health policy in Saudi Arabia. The demonstrated effectiveness of a brief, structured educational intervention in reducing HPV vaccine hesitancy aligns directly with Saudi Vision 2030’s health transformation objectives, which prioritize preventive healthcare, health literacy, and the reduction of non-communicable disease burden across the population. Integrating HPV vaccine education into the curricula of health sciences colleges represents a low-cost, scalable strategy that leverages existing institutional infrastructure to produce a generation of healthcare providers equipped to advocate for vaccination in clinical and community settings. This is particularly relevant in the context of the WHO’s global strategy to accelerate the elimination of cervical cancer, which sets targets of 90% HPV vaccination coverage, 70% cervical cancer screening coverage, and 90% treatment coverage for women with cervical disease by 2030 (8). Saudi Arabia’s current vaccination rate is reported at below 10% among adult females (18, 19), falling dramatically short of this target, underscoring the urgency of scalable demand-generation strategies. Educational interventions targeting healthcare students, who will serve as frontline advocates upon graduation, represent a sustainable upstream investment in achieving these targets. Policy makers should consider mandating HPV vaccine education as a core component of health sciences training and exploring complementary strategies such as provider incentive programs, community awareness campaigns, and school-based vaccination drives to accelerate progress toward national and global elimination goals.

While the intervention achieved significant improvements in knowledge and hesitancy, some limitations must be acknowledged. The quasi-experimental design and reliance on self-reported data introduce potential biases. The absence of a control group means that test–retest familiarity and Hawthorne effects cannot be excluded as contributors to observed gains. The pre-test and post-test were administered on the same day, which may have contributed to response bias or social desirability effects, and the durability of knowledge gains and hesitancy reduction beyond the immediate post-session period remains unknown. Additionally, the study focused on a specific population of healthcare students at a single institution, with over 80% drawn from Applied Medical Sciences alone, limiting representativeness across healthcare disciplines and the generalizability of the findings to other groups. Because questionnaires were anonymous, pre- and post-tests could be linked only through a self-reported identifier. Some responses could not be included in paired analyses due to loss to follow-up or unsuccessful matching; although baseline characteristics were comparable, some potential for selection or attrition bias cannot be excluded. Future research should explore the long-term effects of such interventions and assess their scalability across different demographic groups and regions. Investigating the combined impact of educational programs with other interventions, such as community engagement and provider training, would also provide valuable insights.

## Conclusion

This study demonstrates that targeted educational interventions are effective in improving HPV vaccine knowledge and reducing hesitancy among Saudi healthcare students. By addressing the root causes of hesitancy, such programs have the potential to enhance vaccine uptake and contribute to achieving public health goals. However, to maximize impact, these initiatives should be part of a broader, multi-pronged approach that includes policy support, provider advocacy, and cultural engagement. Future efforts should focus on scaling these interventions and addressing persistent barriers to create a more enabling environment for HPV vaccine acceptance.

## Data Availability

The original contributions presented in the study are included in the article/Supplementary material, further inquiries can be directed to the corresponding author.
