# Supplementary material for: Improving HPV vaccine knowledge and acceptance through educational intervention: a quasi-experimental study of healthcare students in Saudi Arabia
Source: Front Public Health. 2026 Oct 19;14:1940176. doi: 10.3389/fpubh.2026.1940176 (PMC13724403; doi:10.3389/fpubh.2026.1940176)
Supplement: Supplementary file 1 [file Table_1.DOCX]

**Table S1** Baseline characteristics of participants who completed both the pre-test and post-test questionnaires (n = 324) and participants lost to follow-up after the pre-test (n = 31).

|  |  | Completed (324) | Lost to follow up (31) |
| --- | --- | --- | --- |
| **Variable** | **Category** | **N (%)** | |
| Gender | Female | 241 (74.4) | 29 (93.5) |
|  | Male | 83 (25.6) | 2 (6.5) |
| Age (years) | 18–19 | 107 (33.0) | 8 (25.8) |
|  | 20–21 | 180 (55.6) | 22 (71.0) |
|  | ≥22 | 37 (11.4) | 1 (3.2) |
| College | Applied Medical Sciences | 270 (83.3) | 27 (87.1) |
|  | Nursing | 42 (13.0) | 1 (3.2) |
|  | Pharmacy | 9 (2.8) | 1 (3.2) |
|  | Medicine | 2 (0.6) | 0 (0.0) |
|  | Dentistry | 1 (0.3) | 2 (6.5) |
| University year | 1–2 | 125 (38.6) | 20 (64.5) |
|  | 3 | 114 (35.2) | 5 (16.1) |
|  | ≥4 | 78 (24.1) | 6 (19.4) |
| GPA (out of 5) | 4.0–5.0 | 170 (52.5) | 11 (35.5) |
|  | 2.9–3.99 | 134 (41.4) | 15 (48.4) |
|  | <2.9 | 3 (0.9) | 0 (0.0) |
|  | Not disclosed | 17 (5.2) | 5 (16.1) |
| Nationality | Saudi | 320 (98.8) | 31 (100.0) |
|  | Non-Saudi | 4 (1.2) | 0 (0.0) |
| Marital status | Single | 322 (99.4) | 30 (96.8) |
|  | Married | 2 (0.6) | 1 (3.2) |
| Smoking | Non-smoker | 309 (95.4) | 30 (96.8) |
|  | Smoker | 15 (4.6) | 1 (3.2) |
| Heard of HPV vaccine | Yes | 111 (34.3) | 14 (45.2) |
|  | No | 213 (65.7) | 17 (54.8) |
| Vaccine hesitancy | Hesitant | 200 (61.7) | 17 (54.8) |
|  | Not hesitant | 124 (38.3) | 14 (45.2) |

**Table S2** Sensitivity analysis of HPV vaccine knowledge among the 314 linked pre–post pairs using McNemar’s test*.*

| **Statement** | **Pre-intervention %** | **Post-intervention %** | **p value** |
| --- | --- | --- | --- |
| HPV vaccines require three doses | 10.8 | 77.1 | <0.0001 |
| HPV vaccines offer protection against all STIs* | 14.3 | 45.9 | <0.0001 |
| HPV vaccines are most effective if given to people who have never had sex | 18.5 | 79.9 | <0.0001 |
| Someone who has had HPV vaccine cannot develop cervical cancer | 15.3 | 47.5 | <0.0001 |
| HPV vaccines offer protection against most cervical cancers | 22.0 | 79.0 | <0.0001 |
| One of the HPV vaccines offers protection against genital warts | 17.8 | 78.0 | <0.0001 |
| Girls who have had HPV vaccine do not need a Pap smear test when older | 17.5 | 75.2 | <0.0001 |
